# Breast cancer screening practices during a multifaceted crisis: Data from Lebanon

**DOI:** 10.1371/journal.pone.0325604

**Published:** 2025-06-04

**Authors:** Elio Issa, Rachele Lahoud, Aniella Abi-Gerges, Pascale Salameh, Tamina Elias-Rizk

**Affiliations:** 1 Gilbert and Rose-Marie Chagoury School of Medicine, Lebanese American University, Byblos, Lebanon; 2 Department of Radiology, Lebanese American University Medical Center – Rizk Hospital, Achrafieh, Lebanon; 3 Faculty of Pharmacy, Lebanese University, Hadat, Lebanon; 4 Department of Primary Care and Population Health, University of Nicosia Medical School, Nicosia, Cyprus; 5 Institut National de Santé Publique d’Épidémiologie Clinique et de Toxicologie-Liban (INSPECT-LB), Beirut, Lebanon; American University of Beirut, LEBANON

## Abstract

Public health efforts towards breast cancer (BC) prevention have been largely absent from healthcare planning in modern-day Lebanon. Mammography screening campaigns have been present since 2002, but their implementation has been inconsistent in terms of pricing, locations, and the centers involved. In 2020, Lebanon was caught in the whirlwind of the Covid pandemic while facing a brewing economic crisis and a direct hit to the capital’s center of commerce. The impact of the complex situation created by these crises on BC screening remains to be identified, given the high prevalence of BC and the survival benefits provided by early diagnosis. Therefore, we assessed BC screening practices of 400 women aged 35–75 with no prior history of BC from the Lebanese population between January and June 2023. One tenth of participants halted mammography screening, while more than half of participants had continued or improved their BC screening practices after 2020. Women with an unfavorable attitude towards general health check-ups and single participants were more vulnerable to experience change in their BC screenings. Contrarily, women with relatives affected by BC and those financially stable to cover basic needs and more had higher proclivities to undergo BC screening. In this vein, future campaigns should nurture a culture that promotes general health check-ups while still offering financial support. These campaigns should be clearly advertised and communicated to the general public, especially in terms of cost and centers involved. Additional studies are needed to elucidate the determinants that can influence the attitude towards general health check-ups.

## Introduction

Breast cancer (BC) has become the most frequently encountered type of cancer worldwide with over 680,000 deaths in 2020 [1]. In addition to the mortality of the disease, it impacts individuals by the financial burden of healthcare costs, especially in low- and middle-income countries [2]. A recent systematic review and meta-analysis by Ebrahimoghli et al. (2025) found a mean BC screening prevalence of 22.7% among low- and middle-income countries between 2002 and 2023, with great variability in prevalence ranging from 0 to 93.8% [3]. These figures are comparably lower than the prevalence of BC screening in the United States that exceeded 80% in the period extending from 2017 to 2020 [4].

BC was the most frequently diagnosed cancer in Lebanon during the timeframe of 2005–2015, accounting for 20% of all cases of cancer in the country [5]. In 2019, Lebanon had the highest age-standardized incidence rates and age-standardized disability-adjusted life years rates, and was among the top three for age-standardized death rates compared to its neighboring countries [6]. These rankings could be explained by elevated pollution levels in Lebanon (e.g., numerous diesel generators and diesel light duty vehicles) [7]. The literature has described a strong association between atmospheric pollution and BC, with a recent meta-analysis by Gabet *et al.* (2021) implying that a lower exposure to air pollutants may indeed lower the risk of BC [8]. These prominent sequalae highlight the cruciality of implementing low-cost screening measures to ensure early detection of disease and mitigate the high costs associated with treating advanced stages of diseases. Previous studies have identified disease staging as a major contributor to increases in medical expenses with costs increasing by 41% and 165% in patients with regional and distant disease, respectively [9,10]. In addition to the financial benefits of BC screening, there are significant survival benefits. The five-year survival rate of BC patients decreases drastically when the disease is localized (98.8%) versus when it has metastasized (26.3%) [11]. These discrepancies emphasize the importance of early detection for survival, further emphasizing the value of BC screening in improving outcomes.

The majority of low- and middle-income countries lack formal programs for BC screening, and mainly rely on sporadic screening initiatives [12]. Conversely, most European countries have national programs wherein eligible women are systematically screened for BC [13]. The Ministry of Public Health (MoPH) in Lebanon has offered mammographic screening at discounted rates during BC awareness month since 2002. As of 2006, the yearly campaigns were extended to include the three months of the Fall season where mammography is offered at no cost in governmental facilities and at a discounted rate in the private facilities and non-governmental organizations that take part [14,15]. Nevertheless, not all private institutions participated in the campaign, contributing to widely fluctuating – albeit reasonable – pricing during the awareness period [15,16]. Before 2020, healthcare services in Lebanon were covered by either private insurance companies or the MoPH. Private insurance companies offered a variety of payment systems, including strict fee-for-service, fee-for-service coupled with capitation schemes for general practitioners, and plans for co-payment. The MoPH operated on a fee-for-service system with a co-payment by the patient, but outpatient physician visits were not covered [17]. The MoPH used to provide healthcare coverage for half of the Lebanese population [18].

Many events in 2020, including the Lebanese economic crisis, Beirut port explosion, and Covid pandemic, made it a pivotal year that could have potentially affected health screening practices. Lebanon experienced an acute on top of a chronic financial meltdown, among the highest in the world, characterized by a cessation of economic growth and a soaring debt-to-GDP ratio [19]. As a result, Lebanon was demoted to the low- and middle- income group [20]. This deepened the financial barrier to BC screening. Moreover, nearly half of the Lebanese citizens reached beneath the poverty line throughout the multiple stages of lockdown [21,22]. Prices began to soar when the MoPH started to progressively remove national subsidies on several medications with some having nearly tripled and quadrupled. For instance, basic drugs which were once regarded as affordable became inaccessible for most families [23,24].

In addition to the decreased buying power of Lebanese citizens which limited adequate health-seeking behaviors, the healthcare system itself was shifting in a way that further hindered these behaviors. The explosion of the Beirut port – the main hub of commerce in the country – imposed constraints on the importation of medical supplies [25]. The port explosion hindered the flow of medical systems and caused a monopoly of health services by centers with preserved stock and stringent importation deals. The repercussions of the explosion were beyond commercial exchange with massive infrastructure damage to the city [25], worsening the financial resources of surrounding households. Furthermore, Lebanon witnessed the loss of many frontline health care workers with more than 15,000 licensed physicians emigrating to nations where financial opportunities abound, e.g., one health center in the capital reported emigration of around 40% of its emergency health workers [21,26].

Adding to the country’s deteriorating economy, the Covid pandemic was another barrier towards appropriate BC screening practices with the first official case reported in February of 2020 [27]. Lebanese women, like those in many other countries, had to postpone their screening schedules since healthcare professionals directed their efforts towards combatting the outbreak [28]. For instance, many countries halted BC screening in March of 2020, as they considered screening of low priority compared to confronting the pandemic and placing measures to prevent the spread of the virus [28,29].

The Lebanese medical system had run haphazardly in lieu of the aforementioned factors, focusing on urgent care rather than preventive medicine. The MoPH received an influx of applications for healthcare coverage, with about two-thirds of the population seeking assistance. This sudden increase in demand became financially not viable due to a more than 90% reduction in the MoPH budget, which decreased from $300 million to just $20 million [18]. This made the MoPH overwhelmed with providing financial support for more pressing health matters, and leaving awareness and screening interventions on hold. The last mammographic screening campaign was in 2019, and there have been no governmental initiatives since 2020.

The impact of this shift in priorities on BC screening practices, coupled with insufficient BC screening knowledge and unfavorable attitude towards general health check-ups [30], remains to be fully understood and investigated. So far, no study has examined the effects of compounded factors post-2020—such as the Lebanese economic crisis, the Beirut port explosion, and the Covid pandemic—on BC screening practices. This is particularly significant given the lack of a national mammographic screening campaign, which was inconsistently applied, poorly communicated, unevenly distributed across Lebanon, and most crucially, did not mandate serial screening for eligible patients even before 2020. Therefore, this study delves into the BC screening practices amongst women in Lebanon, a low- and middle-income country [20], wrestling with a severe economic crisis, a pandemic, and bursting with cancerous occurrences.

## Materials and methods

### Study population

A minimal sample size of 355 female participants was needed to achieve a 95% confidence level with a five percent margin of error, considering a frequency of 36.2% BC cases according to the latest 2016 data from the Lebanese National Cancer Registry [31]. Our study population comprised a cross-sectional sample of 400 women (aged 35–75) with no history of BC. Participants were recruited from the Lebanese population between January and June 2023 via a 10–15 minute Google Forms questionnaire completed with participants through direct interaction. These participants were encouraged to forward the questionnaire to contacts who fit into our study inclusion criteria. Data was collected upon consent and stored anonymously until a representative sample was reached to ensure external validity. The study was reviewed and granted approval by the Institutional Review Board at our institution under the code number LAUMCRH.TR1.19/Dec/2022.

### Study design

The questionnaire included sections on demographics, attitudes towards general health check-ups, knowledge and perceptions on BC, barriers towards mammographic screening, and BC screening practices. Data based on this questionnaire was already published by Elias-Rizk *et al.* [30], specifically investigating the attitude, knowledge, and barriers towards BC screening in Lebanon using a mix of both the Breast Cancer Screening Beliefs Questionnaire (BCSBQ) [32] and Champion Health Belief Model Scale (CHBMS) validated scales [33]. In this current study, we contemplated to analyze the BC screening practices section of the questionnaire, while focusing on: (1) adherence to BC screening practices and (2) attendance of check-up visits with either a gynecologist or primary care physician, during the periods before and after 2020. The first question assessing BC screening practices offered four response options: “Yes, yearly,” “Yes, every 2 years,” “Yes, more than 2 years interval,” and “No.”

Based on the US Preventive Services Task Force (USPSTF), mammography screening is recommended for women aged 40–74 years every two years [34]. In 2009, the MoPH relied on a compilation of epidemiological data and recommended yearly mammograms starting age 40 if no prior personal or familial history of BC [14]. Accordingly, responses on BC screening practices were categorized as appropriate if participants selected “Yes, yearly,” “Yes, every 2 years,”, and inappropriate if they chose “Yes, more than 2 years interval,” and “No.”

The second question evaluating the practice of health check-up visits was designed as a closed-ended, binary-choice question. The responses of both questions were further categorized based on the shift in health practices before and after 2020: “Diminished” (indicating a negative shift or worsening), “Reinforced” (indicating a positive shift or improvement), “Unchanged - Inappropriate” (no change but initially inappropriate), and “Unchanged - Appropriate” (no change and initially appropriate).

Two additional closed-ended binary questions inquired on the perceived effect of the economic crisis (“*Do you think the economic crisis affected your screening?*”) and the Covid pandemic (“*Do you think the Covid pandemic affected your screening?*”) on BC screening.

### Statistical analysis

Data was analyzed using the SPSS (Statistical Package for Social Sciences) software, version 28.0. Descriptive analysis retrieved frequency and percentage for categorical variables, mean and standard deviation for quantitative variables, after checking for normality. Bivariate analysis used the Chi-square test for dichotomous or multinomial variables, with dependent variables dichotomized.

For the multivariate analysis of appropriate screening practices, a logistic regression with ENTER method was used on dichotomous dependent variables and the model adequacy was checked via Hosmer Lemeshow test. A multivariate MANCOVA was used to compare the means attitude, knowledge and barriers scales among groups (mainly screening appropriateness change), followed by post hoc comparisons with Bonferroni corrections. Independent variables were introduced in the models including baseline clinical variables and sociodemographic factors, after consideration for sample size limitations: age, socioeconomic status quartiles, region, education, marital status, having children, current income, occupation, family history, healthcare coverage, and healthcare professional in the family were included in the model as independent variables. Significance was decided at P-value < 0.05.

## Results

The average age of participants was 49.59 ± 8.71 years. Nearly one fourth of participants had a history of a first-degree relative affected by BC. Close to one fifth of participants believed that their income was insufficient for both essential needs and savings. Most participants had access to BC screening facilities (85.5%), had never screened through national campaigns (79.3%), and were not aware of reduced mammograms pricing during these campaigns (52.2%). Many participants with close connections to the healthcare sector, including those who worked in healthcare (18.2%) and those who had family members working as healthcare professionals (38.2%), had a better attitude towards general health check-ups and fewer barriers to mammographic screening. Additional results on attitude, knowledge, and barriers of BC screening were reported in our previous work by Elias-Rizk *et al*. [30].

Less than half of participants remained consistent in their pursuit of appropriate BC screening practices throughout the crises of 2020 (n = 175, 43.8%), while other participants continued inappropriate adherence to recommended BC screening practices (n = 152, 38%). One tenth of participants stopped their BC screening after 2020 (n = 42, 10.5%), compared to a smaller percentage of participants that started following proper BC screening recommendations after 2020 (n = 31, 7.8%). Similar trends were observed in the percentages of interval change in check-up visits to gynecologist or primary care physicians after 2020, with continued appropriate screening (n = 155, 38.8%) followed by unchanged inappropriate (n = 139, 34.8%), worsened (n = 96, 24.0%), and improved (n = 10, 2.5%) practice.

### Perceived effect of the Lebanese economic crisis on BC screening

Based on bivariate analysis, perceptions regarding the impact of the Lebanese economic crisis on BC screening vary significantly across the area of residency (*P* = < 0.001), education (*P* = 0.041), marital status (*P* = 0.049), income (*P* = < 0.001), having a healthcare professional in the family (*P* = 0.029), easy access to screening facilities (*P* = < 0.001), healthcare coverage (*P* = < 0.001), interval change in both screening status (*P* = < 0.001) and check-up visits (*P* = < 0.001) (Table 1).

**Table 1:** Chi-square of the perceived effect of the Lebanese economic crisis on breast cancer screening.

Variable	Perceived Effect of the Lebanese Economic Crisis on Breast Cancer Screening		*P*-value
	Yes, N (%)	No, N (%)	
**Area of Residency**			
Akkar and North	31 (50.0%)	31 (50.0%)	**<0.001**
Baalbek-Hermel and Bekaa	38 (70.4%)	16 (29.6%)	
Beirut	20 (45.5%)	24 (54.5%)	
Mount-Lebanon	55 (36.7%)	95 (63.3%)	
South	51 (56.7%)	39 (43.3%)	
**Education**			
School Education	90 (56.3%)	70 (43.8%)	**0.041**
University – Bachelor’s Degree	81 (44.8%)	100 (55.2%)	
University – Higher Studies	24 (40.7%)	35 (59.3%)	
**Marital Status**			
Single	24 (44.4%)	30 (55.6%)	**0.049**
Married	156 (47.9%)	170 (52.1%)	
Other	15 (75.0%)	5 (25.0%)	
**Income**			
Not Enough for Basic Needs	49 (65.3%)	26 (34.7%)	**<0.001**
Just Enough for Basic Needs	139 (49.5%)	142 (50.5%)	
Enough for Basic Needs and Savings	7 (15.9%)	37 (84.1%)	
**SES Quartiles**			
≤ .60	66 (52.8%)	59 (47.2%)	0.253
.61 −.80	41 (41.0%)	59 (59.0%)	
.81 - 1.00	55 (52.9%)	49 (47.1%)	
≥ 1.01	33 (46.5%)	38 (53.5%)	
**Occupation**			
Unemployed	54 (50.9%)	52 (49.1%)	0.100
Non-healthcare sector	102 (52.3%)	93 (47.7%)	
Healthcare sector	26 (35.6%)	47 (64.4%)	
Retired	13 (50.0%)	13 (50.0%)	
**Healthcare Professional in the Family**			
No	131 (53%)	116 (47%)	**0.029**
Yes	64 (41.8%)	89 (58.2%)	
**Family History of Breast Cancer in First Degree Relative**			
No	155 (50.5%)	152 (49.5%)	0.206
Yes	40 (43.0%)	53 (57.0%)	
**Access to Screening Facilities (i.e., hospitals, radiology center)**			
No	45 (77.6%)	13 (22.4%)	**<0.001**
Yes	150 (43.9%)	192 (56.1%)	
**Awareness of Reduced Mammography Prices through National Screening Campaign**			
No	104 (49.8%)	105 (50.2%)	0.672
Yes	91 (47.6%)	100(52.4%)	

Residents who were more likely to perceive an effect of the economic crisis on their mammographic screening were: residents of remote and underfunded areas (i.e., Baalbek-Hermel, Bekaa, and the South), females with only a school degree, those who were separated or widowed, participants whose income was insufficient for basic needs, those without a family member in the healthcare system, those without access to nearby screening facilities, self-payers and those relying on defunct insuring parties (e.g., MoPH), or whose check-ups with their primary physician had worsened after 2020.

### Perceived effect of the Covid pandemic on BC screening

Chi-square analysis showed an association of the area of residency (*P* = < 0.001), income (*P* = 0.041), access to screening facilities (*P* = 0.015), interval change in both BC screening status (*P* = < 0.001) and health check-up visits (*P* = < 0.001) with the perceived effect of the Covid pandemic on BC screening (Table 2).

**Table 2:** Chi-square of the perceived effect of the Covid pandemic on breast cancer screening.

Variable	Perceived Effect of the Covid pandemic on Breast Cancer Screening		*P*-value
	Yes, N (%)	No, N (%)	
**Area of Residency**			
Akkar and North	27 (43.5%)	35 (56.5%)	**<0.001**
Baalbek-Hermel and Bekaa	44 (81.5%)	10 (18.5%)	
Beirut	25 (56.8%)	19 (43.2%)	
Mount-Lebanon	64 (42.7%)	86 (57.3%)	
South	43 (47.8%)	47 (52.2%)	
**Education**			
School Education	77 (48.1%)	83 (51.9%)	0.663
University – Bachelor’s Degree	96 (53.0%)	85 (47.0%)	
University – Higher Studies	30 (50.8%)	29 (49.2%)	
**Marital Status**			
Single	23 (42.6%)	31 (57.4%)	0.428
Married	170 (52.1%)	156 (47.9%)	
Other	10 (50.0%)	10 (50.0%)	
**Income**			
Not Enough for Basic Needs	36 (48.0%)	39 (52.0%)	**0.041**
Just Enough for Basic Needs	152 (54.1%)	129 (45.9%)	
Enough for Basic Needs and Savings	15 (34.1%)	29 (65.9%)	
**SES Quartiles**			
≤ .60	58 (46.4%)	67 (53.6%)	0.380
.61 −.80	49 (49.0%)	51 (51.0%)	
.81 - 1.00	60 (57.7%)	44 (42.3%)	
≥ 1.01	36 (50.7%)	35 (49.3%)	
**Occupation**			
Unemployed	49 (46.2%)	57 (53.8%)	0.441
Non-healthcare sector	107 (54.9%)	88 (45.1%)	
Healthcare sector	34 (46.6%)	39 (53.4%)	
Retired	13 (50.0%)	13 (50.0%)	
**Healthcare Professional in the Family**			
No	133 (53.8%)	114 (46.2%)	0.116
Yes	70 (45.8%)	83 (54.2%)	
**Family History of Breast Cancer in First Degree Relative**			
No	153 (49.8%)	154 (50.2%)	0.507
Yes	50 (53.8%)	43 (46.2%)	
**Access to Screening Facilities (i.e., hospitals, radiology center)**			
No	38 (65.5%)	20 (34.5%)	**0.015**
Yes	165 (48.2%)	177 (51.8%)	
**Awareness of Reduced Mammography Prices through National Screening Campaign**			
No	105 (50.2%)	104 (49.8%)	0.831
Yes	98 (51.3%)	93 (48.7%)	
**Healthcare Coverage**			
Self-payer	49 (55.1%)	40 (44.9%)	0.068
Insurance	60 (42.9%)	80 (57.1%)	
Other (CNSS, MoPH, COOP, Army)	94 (55.0%)	77 (45.0%)	

Participants who reported experiencing an impact of the Covid pandemic on their BC screening were: living in Baalbek-Hermel and Bekaa, and the capital city of Beirut, those with income just enough for their basic needs, and those without access to nearby screening facilities. This was also the case for participants who had inappropriate screening before and after 2020 and those whose follow-up with their primary physician had worsened.

### Analysis of change in BC screening appropriateness, with attitudes, knowledge, and barriers

We compared attitudes towards general health check-ups, knowledge about BC screening, and barriers to BC screening among participants whose BC screening improved, worsened, or remained appropriate after 2020, with those whose screening was inappropriate before and after 2020. Those with worsened BC screening after 2020 do not have a worse attitude towards general health check-ups compared to the group with inappropriate screening before and after 2020. Similarly, the fright index – that combined select barriers from the Champion Health Belief Model Scale (CHBMS) – did not change between the latter groups. The attitude towards general health check-ups and fright index of those with worsened mammographic screening are similar to those of the group with appropriate screening before and after 2020. Knowledge about BC screening and barriers towards BC screening does not affect the change in BC screening (Table 3 and S1 for post hoc results). We note that other factors associated with changing to an inappropriate screening were very low income (*P *= 0.003; ORa = 11.801 [2.268; 61.401]), no family history of BC (*p *= 0.046; ORa = 2.409 [1.014; 5.724]), regardless of their attitude, knowledge, barriers or fright. People with no children, very low income and no family history of breast cancer had inappropriate practices overall (Table 3).

**Table 3:** Analysis of change in screening appropriateness, with knowledge, attitudes, and barriers.

Dependent Variable	Screening Change	Mean^**^	Std. Error	95% Confidence Interval	
				Lower	Upper
**Attitudes towards general health check-ups**	Reinforced (improved)	11.162^a^	1.070	9.057	13.267
	Unchanged – inappropriate^*^	13.109^a^	.730	11.675	14.544
	Unchanged – appropriate	**9.813** ^a^	.741	8.356	11.271
	Diminished (worsened)	**10.736** ^a^	.968	8.832	12.640
**Knowledge and perceptions about breast cancer**	Reinforced (improved)	9.854^a^	.925	8.035	11.674
	Unchanged – inappropriate^*^	9.370^a^	.631	8.130	10.611
	Unchanged – appropriate	8.548^a^	.641	7.288	9.808
	Diminished (worsened)	9.152^a^	.837	7.507	10.798
**Barriers to mammographic screening**	Reinforced (improved)	8.534^a^	.846	6.870	10.198
	Unchanged – inappropriate^*^	8.847^a^	.577	7.713	9.982
	Unchanged – appropriate	7.616^a^	.586	6.463	8.768
	Diminished (worsened)	7.675^a^	.765	6.170	9.180
**Additional fright index**	Reinforced (improved)	3.837^a^	.510	2.833	4.841
	Unchanged – inappropriate^*^	4.467^a^	.348	3.783	5.151
	Unchanged – appropriate	**3.531** ^a^	.354	2.836	4.226
	Diminished (worsened)	**3.128** ^a^	.462	2.220	4.036

^a^Covariates appearing in the model are evaluated at the following values: age = 49; Socioeconomic status quartiles, region, education, marital status, having children, current income, occupation, family history, healthcare coverage, and healthcare professional in the family were also included in the model as independent variables.

* Reference category;

** Overall *p*-value <0.001 for attitude, 0.288 for knowledge, 0.061 for barriers, and 0.002 for additional fright index

### Determinants of appropriate BC screening before and after the economic crisis

Based on bivariate analysis, marital status (before 2020: *P* = 0.005, after 2020: *P* = < 0.001), income (both *P* = < 0.001), perceived effect of the Lebanese economic crisis on BC screening (before 2020: *P* = 0.022, after 2020: *P* = 0.058), having a healthcare professional in the family (before 2020: *P* = < 0.001, after 2020: *P* = 0.002), and a first degree relative with BC (both *P* = < 0.001) were associated with BC screening practice both before and after 2020. Conversely, area of residency (*P* = 0.023), and healthcare coverage before (*P* = 0.023) and after 2020 (*P* < 0.001) influenced BC screening practice after 2020 (Table 4).

**Table 4:** Chi-square tests of breast cancer screening practices.

Variable	Breast Screening Practices Before 2020		*P*-value	Breast Screening Practices After 2020		*P*-value
	Appropriate, N (%)	Inappropriate, N (%)		Appropriate, N (%)	Inappropriate, N (%)	
**Area of Residency**						
Akkar and North	29 (46.8%)	33 (53.2%)	0.198	27 (43.5%)	35 (56.5%)	**0.023**
Baalbek-Hermel and Bekaa	26 (48.1%)	28 (51.9%)		19 (35.2%)	35 (64.8%)	
Beirut	25 (56.8%)	19 (43.2%)		22 (50.0%)	22 (50.0%)	
Mount-Lebanon	92 (61.3%)	58 (38.7%)		89 (59.3%)	61 (40.7%)	
South	45 (50.0%)	45 (50.0%)		49 (54.4%)	41 (45.6%)	
**Education**						
School Education	84 (52.5%)	76 (47.5%)	0.237	74 (46.3%)	86 (53.8%)	0.130
University – Bachelor’s Degree	95 (52.5%)	86 (47.5%)		96 (53.0%)	85 (47.0%)	
University – Higher Studies	38 (64.4%)	21 (35.6%)		36 (61.0%)	23 (39.0%)	
**Marital Status**						
Single	19 (35.3%)	35 (64.8%)	**0.005**	17 (31.5%)	37 (68.5%)	**<0.001**
Married	189 (58.0%)	137 (42.0%)		182 (55.8%)	144 (44.2%)	
Other	9 (45.0%)	11 (55.0%)		7 (35.0%)	13 (65.0%)	
**Income**						
Not Enough for basic needs	28 (37.3%)	47 (62.7%)	**<0.001**	29 (26.7%)	55 (73.3%)	**<0.001**
Enough for basic needs	158 (56.2%)	123 (43.8%)		153 (54.4%)	153 (54.4%)	
Enough for basic needs and savings	31 (70.5%)	13 (29.5%)		33 (75.0%)	33 (75.0%)	
**Perceived Effect of the Lebanese Economic Crisis on Breast Cancer Screening**						
No	117 (56.8%)	88 (45.4%)	**0.022**	92 (44.7%)	105 (54.1%)	**0.058**
Yes	89 (43.2%)	106 (54.6%)		114 (55.3%)	89 (45.9%)	
**SES Quartiles**						
≤.60	72 (57.6%)	53 (42.4%)	.793	66 (52.8%)	59 (47.2%)	.970
.61 −.80	54 (54.0%)	46 (46.0%		52 (52.0%)	48 (48.0%)	
.81 - 1.00	53 (51.0%)	51 (49.0%)		53 (51.0%)	51 (49.0%)	
≥1.01	38 (53.5%)	33 (46.5%)		35 (49.3%)	36 (50.7%)	
**Occupation**						
Unemployed	56 (52.8%)	50 (47.2%)	.059	52 (49.1%)	54 (50.9%)	.106
Non-healthcare sector	96 (49.2%)	99 (50.8%)		104 (53.3%)	91 (46.7%)	
Healthcare sector	47 (64.4%)	26 (35.6%)		27 (37.0%)	46 (63.0%)	
Retired	18 (69.2%)	8 (30.8%)		11 (42.3%)	15 (57.7%)	
**Healthcare Professional in the Family**						
No	115 (46.6%)	132 (53.4%)	**<0.001**	112 (45.3%)	135 (54.7%)	**0.002**
Yes	102 (66.7%)	51 (33.3%)		94 (61.4%)	59 (38.6%)	
**Family History of Breast Cancer in First Degree Relative**						
No	151 (49.2%)	156 (50.8%)	**<0.001**	143 (46.6%)	164 (53.4%)	**<0.001**
Yes	66 (71.0%)	27 (29.0%)		63 (67.7%)	30 (32.3%)	
**Healthcare Coverage**						
**Before 2020**						
Self-Payer	28 (48.3%)	30 (51.7%)	.211	30 (51.7%)	28 (48.3%)	**.023**
Insurance	75 (60.5%)	49 (39.5%)		76 (61.3%)	48 (38.7%)	
Other (CNSS, MoPH, COOP, Army)	114 (52.3%)	104 (47.7%)		100 (45.9%)	118 (54.1%)	
**After 2020**						
Self-Payer	28 (48.3%)	30 (51.7%)	0.211	45 (50.6%)	44 (49.4%)	**<.001**
Insurance	75 (60.5%)	49 (39.5%)		90 (64.3%)	50 (35.7%)	
Other (CNSS, MoPH, COOP, Army)	114 (52.3%)	104 (47.7%)		71 (41.5%)	100 (58.5%)	
**Access to Screening Facilities (i.e., hospitals, radiology center)**						
No	22 (37.9%)	36 (62.1%)	**0.007**	17 (29.3%)	41 (70.7%)	**<0.001**
Yes	195 (57.0%)	147 (43.0%)		189 (55.3%)	153 (44.7%)	
**Awareness of Reduced Mammography Prices through National Screening Campaign**						
No	87 (41.6%)	122 (58.4%)	**<0.001**	80 (38.3%)	129 (61.7%)	**<0.001**
Yes	130 (68.1%)	61 (31.9%)		129 (61.7%)	65 (34.0%)	
**Regular Check-Up Visits to a Gynecologist or Primary Care Physician**						
**Before 2020**						
No	43 (28.9%)	106 (71.1%)	**<0.001**	55 (36.9%)	94 (63.1%)	**<0.001**
Yes	174 (69.3%)	77 (30.7%)		151 (60.2%)	100 (39.8%)	
**After 2020**						
No	92 (39.1%)	143 (60.9%)	**<0.001**	77 (32.8%)	158 (67.2%)	**<0.001**
Yes	125 (75.8%)	40 (24.2%)		129 (78.2%)	36 (21.8%)	
**Perceived Effect of the Covid Pandemic on Breast Cancer Screening**						
No	106 (48.8%)	99 (54.1%)	.295	75 (41.0%)	108 (59.0%)	**<.001**
Yes	111 (51.2%)	84 (45.9%)		128 (59.0%)	89 (41.0%)	

The two multivariate analysis models on appropriate screening before and after 2020 revealed that being single (Before 2020: OR = 0.278 [0.128–0.603], *P* = 0.001; After 2020: OR = 0.295 [0.140–0.622], *P* = 0.001), and having an unfavorable attitude towards general health check-ups are associated with inappropriate screening (Before 2020: OR = 0.890 [0.844–0.939], *P* = < 0.001; After 2020: OR = 0.885 [0.840–0.932], *P* = < 0.001), while having a family history of BC is associated with better screening behavior (Before 2020: OR = 2.742 [1.514–4.967], *P* = < 0.001; After 2020: OR = 2.638 [1.489–4.673], *P* = 0.001). Income enough for basic needs and savings became greatly associated with better screening (Table 5).

**Table 5:** Multivariate analysis of features associated with appropriate breast cancer screening practices amongst Lebanese women before and after the economic crisis.

	Appropriate Screening Before 2020				Appropriate Screening After 2020			
	*P*-value	OR	95% C.I.		*P*-value	OR	95% C.I.	
Variables			Lower	Upper		Lower	Upper	
**Age**	**<.001**				**<.001**			
44–49 years vs < 43 years	**<.001**	6.644	3.281	13.455	**<.001**	6.068	3.035	12.132
50–55 years vs < 43 years	**<.001**	10.12	4.845	21.139	**<.001**	4.953	2.480	9.892
56 years and more	**<.001**	8.576	3.787	19.421	**<.001**	4.071	1.877	8.828
**Education level**	0.887				0.448			
University education versus school	0.918	0.969	0.531	1.768	0.217	1.450	0.804	2.614
Higher education versus school	0.711	1.17	0.51	2.685	0.393	1.417	0.637	3.151
**Marital status**	**0.004**				**0.005**			
Other versus married	0.206	0.472	0.147	1.511	0.270	0.522	0.165	1.654
Single versus married	**0.001**	0.278	0.128	0.603	**0.001**	0.295	0.140	0.622
**Income**	**0.008**				**<.001**			
Just enough for basics versus not enough	**0.003**	2.73	1.423	5.239	**<.001**	4.153	2.141	8.054
Enough and more versus not enough	**0.028**	3.026	1.126	8.133	**<.001**	7.956	2.909	21.765
**Occupation**	0.266				0.400			
Works Non health versus does not work	0.625	1.176	0.614	2.251	0.512	0.810	0.431	1.521
Works in Health sector versus does not work	0.074	2.179	0.926	5.124	0.384	1.441	0.634	3.276
Retired versus does not work	0.479	1.5	0.489	4.605	0.943	0.962	0.329	2.814
**Family member is a health professional**	0.063	1.652	0.973	2.804	0.427	1.227	0.740	2.036
**Family history of breast cancer**	**<.001**	2.742	1.514	4.967	**0.001**	2.638	1.489	4.673
**SES quartiles**	0.59				0.803			
Q2 vs Q1	0.401	0.741	0.367	1.493	0.517	0.801	0.409	1.568
Q3 vs Q1	0.201	0.639	0.322	1.27	0.534	0.810	0.416	1.575
Q4 vs Q1	0.802	0.907	0.424	1.941	0.852	1.073	0.514	2.239
**Unfavorable Attitude toward BC screening index**	**<.001**	0.89	0.844	0.939	**<.001**	0.885	0.840	0.932
**Low Knowledge of BC screening index**	0.489	1.025	0.955	1.1	0.442	1.028	0.959	1.101
**Barriers to BC screening index**	0.973	1.002	0.915	1.097	0.966	1.002	0.917	1.095
**Fear of BC screening index**	0.078	0.876	0.756	1.015	0.624	0.965	0.838	1.112

## Discussion

In this study, we investigated the impact of the Lebanese economic crisis and the Covid pandemic on breast mammographic screening practices of Lebanese women. Participants who were single or had an unfavorable attitude towards general health check-ups were more likely to have inappropriate BC screening, compared to those with a positive family history of BC and those with a comfortable financial status enough for basic needs. The latter results echo the findings of a recent systematic review and metanalysis by Mottram *et al*. (2021), wherein appropriate mammographic screening attendance was associated with patients of greater financial security (i.e., greater income and socioeconomic status) [35]. As such, reduced chances of screening are seen in patients with higher levels of financial anxiety [36]. Similar results were also noted in another low- and middle-income country, e.g., Nigeria, as women with lower socioeconomic status used mammographic screening facilities less frequently [37].

Attitude towards general health check-ups affected appropriate BC screening and not knowledge and barriers towards BC screening. This reveals a cultural nuance in Lebanon, where doctor appointments are regarded with higher importance than the actual medical tests and procedures. In Lebanese culture, doctors can sometimes be perceived as authority figures, and the act of visiting them is seen as a pivotal part of the healing process, overshadowing the significance of diagnostic tests. This bolsters the role of physicians in promoting BC screening through fostering a positive attitude towards general health check-ups and continued follow-up. As such, the majority of patients with diabetes and hypertension in Lebanon maintained contact with a physician for health guidance after the events of 2020. Despite financial constraints, many switched to more affordable providers rather than lose access to medical guidance entirely [23], further showcasing that individuals with a positive attitude towards regular check-up visits will find a way to overcome obstacles to healthcare. Given that knowledge is not a determinant of appropriate BC screening, future awareness campaigns should emphasize the importance of undergoing testing as part of general health check-ups, rather than solely focusing on knowledge dissemination. Promoting a favoring attitude towards general health visits can be beneficial for individuals with unchanged inappropriate BC screening behaviors, considering our finding that those with an unfavorable attitude were more likely to have inappropriate BC screening practices. Conversely to the obsolete role of knowledge in our study, a systematic review by Islam et al. (2017) on BC screening practices among six nations of low- and middle-income countries revealed the latter to be a key barrier to screening [38]. This showcases the importance of having a baseline BCSBQ questionnaire to guide nation-specific mammographic campaign to target knowledge towards BC screening versus attitude towards general health check-ups.

Interestingly, variables that impacted screening remained constant before and after 2020; financial status was not differentially associated with change in screening practices before and after 2020, but had a greater impact on BC screening after 2020 compared to before 2020. This could suggest that attitude towards general health check-ups might be the overarching factor that currently influences screening practices most, i.e., despite financial hardship, an individual with favorable general health check-up attitude will still pursue scheduled mammographic screening. In our previous study, most participants had a negative attitude towards general health check-ups [30]. Therefore, additional studies are needed to pinpoint determinants of this attitude to help improve screening for BC and health check-up in general.

Another interesting finding is that the socioeconomic status (SES) quartile was not associated with the perceived economic effect on BC screening, likely since this index represents the overall socioeconomic status of participants, hinting that the financial determinant of BC screening practice is rather the economic status that developed during and not before the crisis. This indicates that not only should future campaigns target attitudes, but efforts should still be made to mitigate financial barriers through providing funding to cover the cost of screening. Moreover, the caveats of current campaigns should be addressed with unified mammography pricing across involved institution and efforts should be made to advertise these details for the sake of transparency. The effect of the economic crisis will only be fully understood once a common baseline positive attitude towards general health check-ups is achieved across the Lebanese population.

Similar to our findings, the study of Mottram et al. (2021) found married participants to have better BC screening practice (Mottram et al., 2021). Nonetheless, given the Lebanese context, some women will still value their family needs as highest priority over theirs (Jamali et al., 2005). This opens a segway for future studies to discuss the priority of health preservation among Lebanese women of different marital status, especially those with children who might bear a mental charge to care for their children at the expense of their health.

The literature supports our finding that patients with a family history of BC are more likely to undergo BC screening themselves [39]. This finding may also be linked to these individuals fostering a positive attitude towards general health check-ups demonstrating stricter adherence to BC screening guidelines. Moreover, consistency in visits to their general practitioners allows close follow-up and initiation of screening appropriately at a younger preventive age. A similar discussion can be carried out for those who have a healthcare worker in the family disseminating proper attitude towards health check-up.

We recorded differences in the perceived effect of the economic crisis on BC screening between areas of residency. In the literature, rural residencies recorded greater diagnoses of advanced disease during periods of economic challenges [9]. The greater economic hurdles present among residents of remote areas can be attributed to the increased fuel costs required to travel to centrally located screening facilities. As such, inaccessible healthcare facilities were reported to significantly lower appropriate screening practices [39]. Additionally, remote areas in Lebanon are predominantly agricultural with lower incomes, making it challenging to keep up with the rising prices of screening and doctor visits. Moreover, the geographic distribution of our cohort affected their perceived effect of the Covid pandemic on BC screening. This discrepancy may partly be explained by Lebanon’s unevenly distributed population across urban and rural regions, the extent of the pandemic in each region, and the different isolation measures deployed in each region. Indeed, as the pandemic unfolded, urban centers saw the largest number of positive Covid cases versus more remote areas during June of 2020 [40]. Furthermore, areas were differently affected due to the implementation of strict health-related regulations, e.g., screening all individuals returning from travel or exhibiting symptoms, the nationwide curfew restricting transport, coupled with the limitations of public transport [40].

Access to screening facilities for our participants was limited with 65% of people having no access to screening facilities, and hence being further affected by the pandemic. During this time, numerous healthcare facilities had to concentrate on caring for patients affected by Covid and diverting resources and medical personnel towards combating this disease. For instance, the number of standard cancer screenings, including BC screenings, dropped by 89.2% through May 2020 in the United States [41,42]. In a study conducted in a Middle eastern central university hospital, 73% of the breast unit activity took place during the pandemic [43]. These results may be attributed to the national curfew limiting access to planned care, as well as the lack of screening campaigns and screening availability during this period [40]. In addition, the fear of contracting the virus could have impacted women’s willingness to visit a hospital or medical center. In a study conducted on more than 1800 Lebanese participants, about one third were afraid of the disease and 23.8% scared of dying from this virus [44]. Given the strict disease containment measures, from following curfew hours to wearing personal protective equipment, Lebanese citizens were reluctant to leave their house for health concerns. This translated in a significant 47.2% drop in ED visits during the first 90 days of the pandemic [45].

Despite the major financial burden of the Covid quarantine reported amongst Lebanese women, due to the nationwide shutdown and job layoffs [40], income was not associated with the perceived effect of the Covid pandemic on BC screening. As previously discussed, the implications of constraints caused by the Covid pandemic on income cannot be fully elucidated unless we overcome improper attitude on BC screening as a barrier to mammographic pursuits.

A significant interval change in the regular checkup visits was noted, with 69.8% of women with diminished regular check-ups (n = 67) reporting being affected by the pandemic. Indeed, many people avoided going to clinics, mainly of the public sector, even for fully funded services, out of fear of contracting the virus [40]. Nonetheless, 45.2% reported an unchanged-appropriate status (n = 70) affected by the pandemic, that could be explained by the fear and death anxiety created by the pandemic, as well as the reported increased in somatic symptoms (e.g., increased heart rates or palpitations and increased sleep disturbances) as they thought about the virus [44].

Despite going through a unique set of circumstances, our findings can be extrapolated beyond Lebanese women. Financial hurdles remain true for low- and middle-income countries that face their own set of obstacles hindering their financial stability. Beyond the financial barriers, the main obstacle to BC screening was attitude towards general health check-ups, which will remain true for every other country displaying a lack of favorable general health check-ups.

### Limitation

This is the first study that investigated the impact of compounded challenges from the Lebanese economic crisis to the Covid pandemic on BC screening practices amongst Lebanese females. We were not able to isolate the effect of either challenges on BC screening as they both coincided in time and can be interrelated. Our cohort was chosen from the team’s contacts to achieve a snowball collection through their contacts, which in theory might constrict representation of the Lebanese population [46]. Nonetheless, our sample ended up diverse in demographics which supports the external validity of this study. We also acknowledge potential subjective interpretation of the questionnaire, related to a possible information bias. Residual confounding bias might have influenced our observed associations during multivariate analysis due to potential variables we did not account for.

### Recommendation for future research

The impact of delayed mammographic screening on disease burden, including staging and metastasis at time of diagnosis, should be assessed especially in those who had a shift towards inappropriate screening practice and received a BC diagnosis.

## Conclusion

The events of the year 2020 from the Lebanese economic crisis to the Covid pandemic have exposed the fragility of the Lebanese health system and escalated preexisting healthcare vulnerabilities, further leading to significant disruptions in screening services and a decline in participation rates.

The main obstacle to improving screening practices is the need to cultivate a culture that values general health check-ups. The determinants that foster a positive attitude towards seeking medical care should be identified and leveraged in future campaigns. Even if individuals were not differentially affected by the events of 2020, their financial status before the pandemic still impacted screening practices, hence the inevitability of providing monetary support during future campaigns. Clear advertisement of centers involved in the campaign and unified mammography fees are recommended.

Our results highlight the pressing need for a structured plan regarding women’s health in emergency situations. Our plan could ensure continuity of care for Lebanese women, in order to reduce the heavy burden and long-term effects of BC – a major public health enemy in Lebanon.

## Supporting information

S1 Table Pairwise comparisons. (DOCX)
