# Supplementary material for: Breast cancer screening practices during a multifaceted crisis: Data from Lebanon
Source: PLoS One. 2025 Jun 4;20(6):e0325604. doi: 10.1371/journal.pone.0325604 (PMC12136467; doi:10.1371/journal.pone.0325604)
Supplement: S1 Table — (DOCX) [file pone.0325604.s001.docx]

**Table S1**. Pairwise comparisons

| **Dependent Variable** | **(I) Screening change** | **(J) Screening change** | **Mean Difference (I-J)** | **Std. Error** | ***P*-value^b^** | **95% Confidence Interval for Difference^b^** | |
| --- | --- | --- | --- | --- | --- | --- | --- |
|  |  |  |  |  |  | **Lower Bound** | **Upper Bound** |
| **Attitude towards general health check-ups** | **Became appropriate after** | **Inappropriate before and after** | -1.948 | .964 | .264 | -4.505 | .610 |
|  |  | **Appropriate before and after** | 1.348 | .965 | .979 | -1.211 | 3.908 |
|  |  | **Became inappropriate after** | .426 | 1.164 | 1.000 | -2.663 | 3.515 |
|  | **Inappropriate before and after** | **Became appropriate after** | 1.948 | .964 | .264 | -.610 | 4.505 |
|  |  | **Appropriate before and after** | 3.296^*^ | .608 | **<.001** | 1.683 | 4.908 |
|  |  | **Became inappropriate after** | 2.374^*^ | .868 | **.039** | .071 | 4.676 |
|  | **Appropriate before and after** | **Became appropriate after** | -1.348 | .965 | .979 | -3.908 | 1.211 |
|  |  | **Inappropriate before and after** | -3.296^*^ | .608 | **<.001** | -4.908 | -1.683 |
|  |  | **Became inappropriate after** | -.922 | .850 | 1.000 | -3.178 | 1.333 |
|  | **Became inappropriate after** | **Became appropriate after** | -.426 | 1.164 | 1.000 | -3.515 | 2.663 |
|  |  | **Inappropriate before and after** | -2.374^*^ | .868 | **.039** | -4.676 | -.071 |
|  |  | **Appropriate before and after** | .922 | .850 | 1.000 | -1.333 | 3.178 |
| **Knowledge on breast cancer screening** | **Became appropriate after** | **Inappropriate before and after** | .484 | .833 | 1.000 | -1.727 | 2.694 |
|  |  | **Appropriate before and after** | 1.306 | .834 | .709 | -.906 | 3.519 |
|  |  | **Became inappropriate after** | .702 | 1.007 | 1.000 | -1.968 | 3.371 |
|  | **Inappropriate before and after** | **Became appropriate after** | -.484 | .833 | 1.000 | -2.694 | 1.727 |
|  |  | **Appropriate before and after** | .822 | .526 | .711 | -.571 | 2.216 |
|  |  | **Became inappropriate after** | .218 | .750 | 1.000 | -1.772 | 2.208 |
|  | **Appropriate before and after** | **Became appropriate after** | -1.306 | .834 | .709 | -3.519 | .906 |
|  |  | **Inappropriate before and after** | -.822 | .526 | .711 | -2.216 | .571 |
|  |  | **Became inappropriate after** | -.604 | .735 | 1.000 | -2.554 | 1.345 |
|  | **Became inappropriate after** | **Became appropriate after** | -.702 | 1.007 | 1.000 | -3.371 | 1.968 |
|  |  | **Inappropriate before and after** | -.218 | .750 | 1.000 | -2.208 | 1.772 |
|  |  | **Appropriate before and after** | .604 | .735 | 1.000 | -1.345 | 2.554 |
| **Barriers towards mammographic screening** | **Became appropriate after** | **Inappropriate before and after** | -.314 | .762 | 1.000 | -2.335 | 1.708 |
|  |  | **Appropriate before and after** | .918 | .763 | 1.000 | -1.106 | 2.941 |
|  |  | **Became inappropriate after** | .859 | .921 | 1.000 | -1.583 | 3.300 |
|  | **Inappropriate before and after** | **Became appropriate after** | .314 | .762 | 1.000 | -1.708 | 2.335 |
|  |  | **Appropriate before and after** | 1.232 | .481 | .065 | -.043 | 2.506 |
|  |  | **Became inappropriate after** | 1.172 | .686 | .530 | -.648 | 2.992 |
|  | **Appropriate before and after** | **Became appropriate after** | -.918 | .763 | 1.000 | -2.941 | 1.106 |
|  |  | **Inappropriate before and after** | -1.232 | .481 | .065 | -2.506 | .043 |
|  |  | **Became inappropriate after** | -.059 | .672 | 1.000 | -1.842 | 1.724 |
|  | **Became inappropriate after** | **Became appropriate after** | -.859 | .921 | 1.000 | -3.300 | 1.583 |
|  |  | **Inappropriate before and after** | -1.172 | .686 | .530 | -2.992 | .648 |
|  |  | **Appropriate before and after** | .059 | .672 | 1.000 | -1.724 | 1.842 |
| **Fear of breast cancer screening** | **Became appropriate after** | **Inappropriate before and after** | -.630 | .460 | 1.000 | -1.850 | .589 |
|  |  | **Appropriate before and after** | .306 | .460 | 1.000 | -.915 | 1.527 |
|  |  | **Became inappropriate after** | .709 | .555 | 1.000 | -.764 | 2.182 |
|  | **Inappropriate before and after** | **Became appropriate after** | .630 | .460 | 1.000 | -.589 | 1.850 |
|  |  | **Appropriate before and after** | .936^*^ | .290 | .**008** | .167 | 1.705 |
|  |  | **Became inappropriate after** | 1.339^*^ | .414 | **.008** | .241 | 2.437 |
|  | **Appropriate before and after** | **Became appropriate after** | -.306 | .460 | 1.000 | -1.527 | .915 |
|  |  | **Inappropriate before and after** | -.936^*^ | .290 | **.008** | -1.705 | -.167 |
|  |  | **Became inappropriate after** | .403 | .406 | 1.000 | -.673 | 1.479 |
|  | **Became inappropriate after** | **Became appropriate after** | -.709 | .555 | 1.000 | -2.182 | .764 |
|  |  | **Inappropriate before and after** | -1.339^*^ | .414 | **.008** | -2.437 | -.241 |
|  |  | **Appropriate before and after** | -.403 | .406 | 1.000 | -1.479 | .673 |
| Based on estimated marginal means | | | | | | | |
| ^*^. The mean difference is significant at the .05 level. | | | | | | | |
| ^b^. Adjustment for multiple comparisons: Bonferroni. | | | | | | | |
